# Probing horseradish peroxidase catalyzed degradation of azo dye from tannery wastewater

**DOI:** 10.1186/2193-1801-2-341

**Published:** 2013-07-24

**Authors:** Sadhanandam Preethi, Ayyappan Anumary, Meiyazhagan Ashokkumar, Palanisamy Thanikaivelan

**Affiliations:** Advanced Materials Laboratory, Center for Leather Apparel & Accessories Development, Central Leather Research Institute (Council of Scientific and Industrial Research), Adyar, Chennai, 600 020 India

**Keywords:** Biocatalysis, Immobilization, Wastewater, Kinetics, Recycling

## Abstract

Biocatalysis based effluent treatment has outclassed the presently favored physico-chemical treatments due to nil sludge production and monetary savings. Azo dyes are commonly employed in the leather industry and pose a great threat to the environment. Here, we show the degradation of C. I. Acid blue 113 using horseradish peroxidase (HRP) assisted with H_2_O_2_ as a co-substrate. It was observed that 0.08 U HRP can degrade 3 mL of 30 mg/L dye up to 80% within 45 min with the assistance of 14 μL of H_2_O_2_ at pH 6.6 and 30°C. The feasibility of using the immobilized HRP for dye degradation was also examined and the results show up to 76% dye degradation under similar conditions to that of free HRP with the exception of longer contact time of 240 min. Recycling studies reveal that the immobilized HRP can be recycled up to 3 times for dye degradation. Kinetics drawn for the free HRP catalyzed reaction marked a lower *K*_m_ and higher *V*_max_ values, which denotes a proper and faster affinity of the enzyme towards the dye, when compared to the immobilized HRP. The applicability of HRP for treating the actual tannery dye-house wastewater was also demonstrated.

**Electronic supplementary material:**

The online version of this article (doi:10.1186/2193-1801-2-341) contains supplementary material, which is available to authorized users.

## Background

Synthetic dyes are extensively used in a variety of industries including leather and textiles. Conversion of skin into leather generates huge quantity of wastewater comprising a mixture of biogenic matter of skins and a large variety of organic and inorganic chemicals. Wastewater from tanneries usually contains high concentrations of dyes, neutral salts, aliphatic and aromatic polymeric substances as well as poly-phenols (Murugananthan et al. 2004). Acid azo dyes are much frequently used and are potentially toxic if left untreated and there is a high risk to the natural flora (Mohan et al. 2005). The chemical constituents of the dye are mainly phenolic compounds. Since these dye molecules are often toxic and hard to degrade in the conventional wastewater treatment systems, urgency for proper treatment of the colored effluent is required.

Although a variety of physico-chemical treatments such as adsorption, precipitation, chemical degradation, electrochemical, photochemical, etc. is available to decolorize the dye-house wastewater, they have inherent disadvantages such as demand for an external reagent, large sludge generation, and are expensive and tedious (Gozmen et al. 2009; Sauer et al. 2006; Rodriguez et al. 2010; Lachheb et al. 2002). Hence, alternative treatment processes based on biotechnological principles have gained popularity in recent years (Palmieri et al. 2005; Ollikka et al. 1993). Enzymatic treatment systems are simpler and easy to operate in comparison to the microbial treatment systems (Mohan et al. 2005; Kandelbauer et al. 2004). The catalytic action of enzymes is efficient, selective, have higher reaction rates and require mild reaction conditions compared to chemical catalysts. Oxidative enzymes such as lignin peroxidase, horseradish peroxidase (HRP), manganese peroxidase and phenoloxidase (laccase) are extensively employed to remove color from effluent by oxidative degradation of colored compounds and to degrade toxic polyphenols, polyaromatic hydrocarbons, polychlorinated biphenyls, etc. (Mohan et al. 2005; Ollikka et al. 1993; Kandelbauer et al., 2004; Regalado et al. 2004). Reduction of peroxides at the expense of electron donating substrates makes peroxidases useful in oxidative breakdown of synthetic azo dyes (Regalado et al. 2004).

HRP (EC: 1.11.1.7) is known to degrade a wide spectrum of aromatic compounds such as phenols, anilines as well as dyes in the presence of H_2_O_2_ (Mohan et al. 2005; Wagner and Nicell 2001; Ulson de Souza et al. 2007; Onder et al. 2011; Arslan 2011; Gholami-Borujeni et al. 2011). The enzyme has relatively high thermal stability and wide distribution (Regalado et al. 2004). H_2_O_2_ helps to oxidize the enzyme into a catalytically active form that is capable of reacting with the phenolic contaminant. Peroxidases act by generating free-radical compounds followed by spontaneous polymerization, the polymers can then be removed from the aqueous phase (Wagner and Nicell 2001). The use of immobilized HRP for treating effluent is becoming popular since it can offer long lifetime, stability and recyclability (Mohan et al. 2005; Arslan 2011; Alemzadeh and Nejati 2009). However, appropriate selection of encapsulation material specific to the enzyme and optimization of process conditions is still a challenge (Mohan et al. 2005). Here, we show the ability of HRP both in its free and immobilized form to decolorize an industrially important azo dye, C.I. Acid blue 113. Various parameters such as pH, temperature, contact time, H_2_O_2_ and HRP concentration, and dye concentration have been investigated to optimize the treatment conditions. Further, the immobilized HRP performance was evaluated in the process of dye removal along with its recyclability. The enzyme kinetics and ability to treat real tannery effluent were also examined.

## Experimental

### Materials

HRP was procured from M/s Medox Biotech Pvt. Ltd., Chennai. 1 μL of the enzyme solution contained 10 U. Specific activity was >250 Pur U/mg. 0.1 M phosphate buffer was prepared using sodium, potassium salts and their respective bases and used to dilute the enzyme such that 2 μL solution contains 0.08 U of HRP. H_2_O_2_ used was of analytical grade (30% w/v). The buffers and other chemical reagents used in this study were of laboratory grade. Commercial C. I. Acid Blue 113 was procured from the Leather Processing Division of Central Leather Research Institute, Chennai and its chemical properties are presented in Table 1. Standard solutions of the dye were prepared by dissolving a known quantity of C.I. Acid Blue113 in distilled water. The pH of the dye solution was 6.6. λ_max_ for C.I.Acid Blue 113 was found to be 566 nm and the molar extinction coefficient was calculated as 16484.3 l mol^-1^ cm^-1^ using Beer-Lambert law.

**Table 1:** **Chemical properties of C.I. Acid Blue 113 (Colour index number 26360)**

Properties	Value
Molecular formula	C_32_H_21_N_5_Na_2_O_6_S_2_
Molecular structure	
Molecular weight	681.65
Water solubility	40 mg/mL
Chromophore	Diazo
λ_max_	566 nm

### Optimization of process parameters for the removal of color using free HRP

Optimization of various process parameters such as pH, temperature, contact time, H_2_O_2_, HRP concentration and dye concentration was carried out. Batch reactions were conducted at room temperature maintained at 30°C using borosilicate vials of 15 mL capacity. Each reaction mixture consisted of 3 mL of 30 mg/L dye, 14 μL of H_2_O_2_ and 0.08 U HRP for all the test series except during dye, H_2_O_2_ and HRP optimization. The vials containing the reaction mixture were not subjected to stirring or any kind of agitation. The reaction mixture displayed a pH of 6.6. Dye decolorization was measured spectrophotometrically using a UV-Visible spectrophotometer (Model UV-160A; Shimadzu) based on the absorbance at 566 nm (λ_max_). Similar experiments were performed by varying the contact time (0, 15, 30, 45, 60, 75, 90, 105, 120 min), pH (4, 5, 6.6, 8, 9, 10), HRP concentration (0.02, 0.04, 0.06, 0.08, 0.10, 0.12 U), H_2_O_2_ concentration (2, 4, 6, 8, 12, 14, 16, 20 μL), temperature (20, 30, 40, 50, 60°C) and dye concentration (20, 25, 30, 35, 40, 45, 50 mg/L). Experiments were performed in triplicate and the mean values of the results are presented along with error bars. For the pH optimization studies, diluted HCl and NaOH were used to adjust the pH of the samples. The percentage color removal was calculated using the following equation.

Where *A*_*0*_ is the absorbance of the dye solution before enzymatic treatment at 566 nm *A*_*T*_ is the absorbance of the dye solution after enzymatic treatment at 566 nm.

### Immobilization of HRP

0.08 U of HRP was added to 1 mL of 0.02 g/mL sodium alginate solution and mixed well. The mixture was dropped into 50 mL of 0.1 M calcium chloride solution through a pipette to form beads while the bottom of the flask was continuously shaken. The beads were left undisturbed in calcium chloride solution to attain stability at 30°C for 40 min. The beads were then filtered and used for further studies.

### Optimization of process parameters for the removal of color using immobilized HRP

Optimization of various process parameters such as contact time, temperature, H_2_O_2_ and HRP concentration was carried out. Batch reactions were conducted at room temperature maintained at 30°C using borosilicate vials of 15 mL capacity. Each reaction mixture consisted of 3 mL of 30 mg/L dye, 14 μL of H_2_O_2_ and 0.08 U of immobilized HRP for all the test series except during H_2_O_2_ and HRP optimization. The vials containing the reaction mixture were not subjected to stirring or any kind of agitation. The reaction mixture displayed a pH of 6.6. Dye decolorization was measured spectrophotometrically using a UV-Visible spectrophotometer (Model UV-160A; Shimadzu) based on the absorbance at 566 nm (λ_max_). Similar experiments were performed by varying the contact time (0, 15, 30, 60, 90, 130, 180, 210, 240, 270 min), immobilized HRP concentration (0.02, 0.04, 0.06, 0.08, 0.10, 0.12 U), H_2_O_2_ concentration (4, 8, 12, 14, 16, 20 μL) and temperature (4, 30, 50°C). Experiments were performed in triplicate and the mean values of the results are presented along with error bars.

### Recycling studies for the removal of color using immobilized HRP

Each reaction mixture consisted of 3 mL of 30 mg/L dye, 14 μL of H_2_O_2_ and 0.08 U of immobilized HRP were kept for different time intervals from 60 to 240 min at 30°C and pH 6.6 for the first cycle experiments. The extent of decolorization after each point of time was analyzed spectrophotometrically as above. Experiments were performed in triplicate and the mean values of the results are presented along with error bars. After the experiments, the beads were collected, washed and reused for the second and third cycle of experiments.

### Enzyme kinetics

The kinetic experiments for the free HRP were performed by varying the concentration of dye (20, 30, 40 and 50 mg/L) and the contact time (15, 30, 45, 60, 75 min) using constant free enzyme and H_2_O_2_ concentration under the optimum conditions at pH 6.6 and 30°C (see Additional file 1). Similarly, the kinetic experiments for the immobilized HRP were performed by varying the concentration of dye (20, 30, 40 and 50 mg/L) and the contact time (25, 85, 125, 200 min) using constant immobilized enzyme and H_2_O_2_ concentration under the optimum conditions at pH 6.6 and 30°C (see Additional file 1). The maximum rate of decolorization reaction (*V*_max_) and Michaelis-Menten constant (*K*_m_) of free as well as immobilized HRP were determined by linear regression and the Lineweaver – Burk plots.

## Results and discussion

### Optimization of process parameters using free HRP

**Figure 1:**
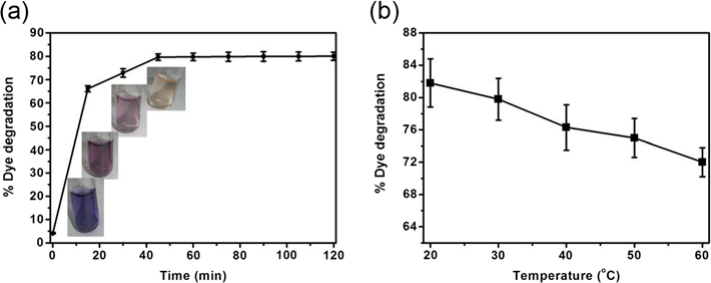
**Effect of (a) time and (b) temperature on the free HRP catalyzed color removal.** Inset in Figure 1**(a)** shows the photographs of dye solution during the course of HRP treatment up to 45 min.

Initial experiments were performed to determine the optimum contact time required to degrade the dye using free HRP. A series of experiments with 3 mL of 30 mg/L dye, 14 μL of H_2_O_2_ and 0.08 U free HRP were conducted for different time intervals up to 120 min at 30°C and pH 6.6. For every 15 min, one vial was analyzed for the dye concentration using UV-Visible spectrophotometer as described above. It is seen from Figure 1a that there is a significant increase in the degradation of dye up to 45 min of contact time. After 45 min, no substantial improvement in the degradation of dye was observed. Hence, subsequent experiments were carried out for 45 min as contact time. Temperature is another important parameter governing the action of enzymes. Experiments were carried out using 3 mL of 30 mg/L dye, 14 μL of H_2_O_2_ and 0.08 U free HRP and kept for different temperatures from 20 to 60°C for 45 min at pH 6.6. The extent of degradation of dye as a function of temperature is shown in Figure 1b. It is evident that the maximum color removal is achieved at lower temperature. It has been shown that free HRP has maximum activity at temperatures as low as 5°C (Shi et al. 2011). Although we found that the maximum color removal is achieved at 20°C, we have carried out all further experiments in this study at room temperature (30°C) considering the practical limitations on the application of this technology.

**Figure 2:**
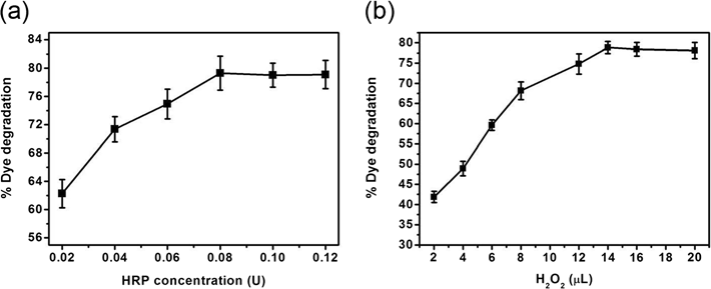
**Effect of (a) enzyme and (b) H**_**2**_**O**_**2**_**concentration on the free HRP catalyzed color removal.**

The relation between the concentration of enzyme and substrate is important in achieving maximum catalytic activity of the enzyme. In order to find the optimum enzyme dosage for the chosen dye concentration, various experiments were performed with 3 mL of 30 mg/L dye and 14 μL of H_2_O_2_ with varying dosage of free HRP from 0.02 to 1.12 U for 45 min at pH 6.6 and 30°C and the results are shown in Figure 2a. It is seen that the degradation of color is increasing as the concentration of HRP increases and attain saturation at 0.08 U. Further increase in the dosage of HRP did not result in pronounced color removal. Hence, optimum HRP concentration is finalized as 0.08 U and used in all the subsequent experiments. To find the optimum H_2_O_2_ concentration, various experiments were performed with 3 mL of 30 mg/L dye and 0.08 U free HRP with varying concentration of H_2_O_2_ from 2 to 20 μL for 45 min at pH 6.6 and 30°C and the results are shown in Figure 2b. It is seen that the degradation of dye is very low (~42%) at low concentration of H_2_O_2_. However, a further increase in concentration of H_2_O_2_ resulted in pronounced increase in the degradation of dye reaching up to 78%, which underscores the role of H_2_O_2_ on the catalytic activity of HRP. Saturation of dye degradation is noted after 14 μL H_2_O_2_, which was selected as an optimum concentration and used in all the subsequent experiments.

**Figure 3:**
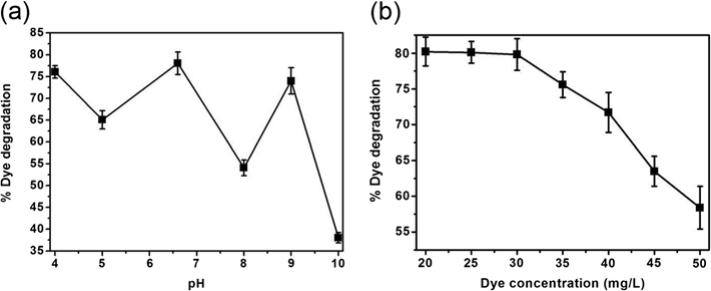
**Effect of (a) pH and (b) dye concentration on the free HRP catalyzed color removal.**

pH is an important factor for the activity of any enzyme and in particular for HRP. To find the optimum pH for the HRP to degrade the dye, various experiments were performed with 3 mL of 30 mg/L dye, 14 μL of H_2_O_2_ and 0.08 U free HRP with varying pH conditions from 4 to 10 for 45 min at 30°C and the results are shown in Figure 3a. It is seen that the free HRP exhibits higher percentage of color removal at various pH values such as 4, 6.6 and 9. It is known that free HRP has higher activity on a wide pH range (Onder et al. 2011; Shi et al. 2011). Hence, we have chosen pH 6.6 as the optimum pH and used in all the subsequent experiments. Concentration of the substrate is another important factor, which will affect the performance of the enzymatic reaction. To determine the optimum substrate concentration, experiments were performed with 14 μL of H_2_O_2_ and 0.08 U free HRP with 3 mL of different dye concentrations from 20 to 45 mg/L for 45 min at pH 6.6 and 30°C and the results are shown in Figure 3b. As we can see from Figure 3b, the degradation of dye is higher at low concentration of dye from 20 to 30 mg/L. Beyond which, the degradation of dye is decreasing abruptly as the concentration of dye increases. Hence, the optimum substrate concentration was fixed as 30 mg/L for all the subsequent experiments.

### Optimization of process parameters using immobilized HRP

**Figure 4:**
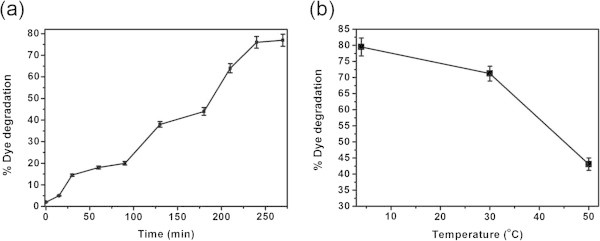
**Effect of (a) time and (b) temperature on the biocatalytic color removal using immobilized HRP.**

**Figure 5:**
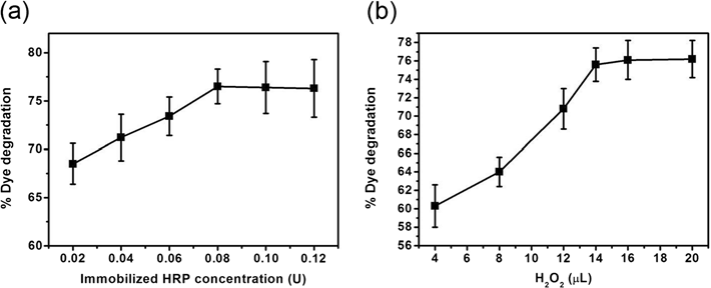
**Effect of (a) enzyme and (b) H**_**2**_**O**_**2**_**concentration on the biocatalytic color removal using immobilized HRP.**

Immobilization of enzyme is an important technique to reduce the cost of enzymatic processes as it helps in recovery and recycling of entrapped enzymes for more than one cycle of reaction. Here, we have immobilized the free HRP in calcium alginate beads and experiments were performed to optimize the process parameters for the application of immobilized HRP for decolorizing C.I. Acid Blue113. In order to optimize the contact time, experiments with 3 mL of 30 mg/L dye, 14 μL of H_2_O_2_ and 0.08 U immobilized HRP were kept for different time intervals up to 270 min at 30°C and pH 6.6. The extent of decolorization as a function of time is shown in Figure 4a. A gradual increase in the degradation of dye is noticed as the contact time increases. In comparison to free HRP (45 min), significantly high contact time (240 min) is required for immobilized HRP to degrade the dye appreciably. This may be due to the entrapment of HRP in the calcium alginate beads. To find the optimum temperature, various experiments were performed with 3 mL of 30 mg/L dye, 14 μL of H_2_O_2_ and and 0.08 U immobilized HRP at different temperatures from 4 to 50°C for 240 min at pH 6.6. The results are shown in Figure 4b. Akin to free HRP, the extent of dye degradation is significantly reduced as the temperature of the reaction increases. However, all the further experiments were conducted at room temperature (30°C) considering the practical feasibility. To find the optimum immobilized HRP concentration for degrading the dye, various experiments were performed with 3 mL of 30 mg/L dye and 14 μL of H_2_O_2_ with varying immobilized HRP concentration for 240 min at pH 6.6 and 30°C and the results are shown in Figure 5a. It is seen that the extent of dye degradation increases as the concentration of immobilized HRP increases reaching saturation at 0.08 U. This value is similar to that observed for free HRP. In order to find the optimum H_2_O_2_ concentration, experiments were performed with 3 mL of 30 mg/L dye and 0.08 U immobilized HRP with varying concentration of H_2_O_2_ from 4 to 20 μL for 240 min at pH 6.6 and 30°C and the results are shown in Figure 5b. It is observed that the dye degradation reaches maximum at 14 μL H_2_O_2_ beyond which there is no significant increase. This trend is also not very different from free HRP.

### Recyclability of immobilized HRP

**Figure 6:**
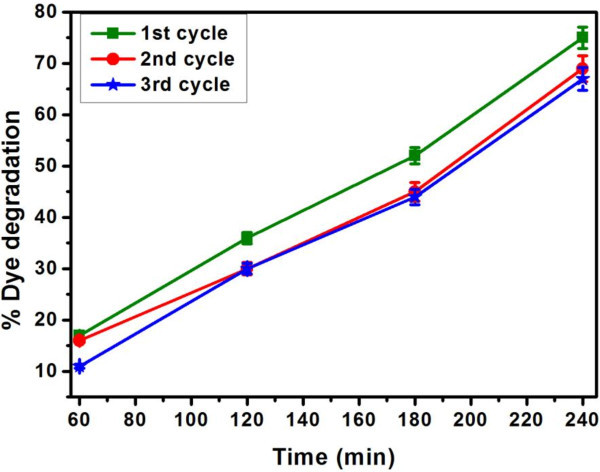
**Recyclability of immobilized HRP for the biocatalytic color removal for 3 cycles as a function of contact time.**

An important advantage of immobilized enzymes is their recyclability in the process thereby reducing the cost of treatment. Hence, we have examined the recycling ability of immobilized HRP for the color removal and the results are shown in Figure 6. It is seen that immobilized HRP can be recycled for at least 3 cycles without losing much of its activity. The color removal efficiency of immobilized HRP followed similar pattern for all the 3 cycles as a function of time. There seems to be a marginal reduction in the extent of decolorization during the 2nd and 3rd cycle of experiments. Hence, it is demonstrated that the immobilized HRP can reduce the cost of color removal at industrial level applications.

### Kinetics of free and immobilized HRP

**Figure 7:**
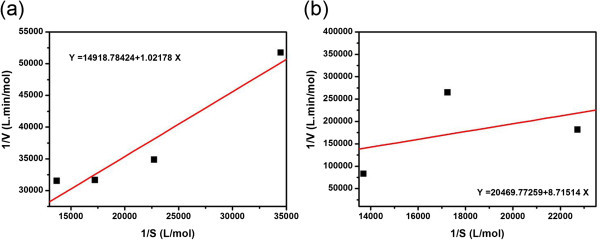
**Lineweaver-Burk plot for the biocatalytic degradation of the dye using (a) free and (b) immobilized HRP.**

The kinetics were obtained by observing the degradation of different concentrations of dye at specified time intervals using optimized H_2_O_2_ concentration and free as well as immobilized HRP concentration under the optimum conditions (at pH 6.6, 30°C). Lineweaver-Burk plots (double reciprocal plot) were made for both free and immobilized HRP as shown in Figure 7. The slope (*K*_m_/*V*_max_) and intercept (1/*V*_max_) values were obtained from the plots in order to calculate the apparent Michaelis-Menten constant (*K*_m_) as well as the maximum rate of the reaction (*V*_max_). The calculated value of the apparent *K*_m_ for free HRP is 0.068 mmol/l, which is lower than immobilized HRP (0.425 mmol/l). Also, the calculated value of *V*_max_ for free HRP is 0.067 mmol/l.min, which is higher than immobilized HRP (0.048 mmol/l.min). In general, lower *K*_m_ means better binding of the enzyme to its substrate and higher *V*_max_ indicates faster reaction. Kinetics calculated in this study shows higher affinity of free HRP (low *K*_m_) for the degradation of the dye. Higher apparent *K*_m_ and lower *V*_max_ values obtained for the immobilized HRP suggest that the immobilization of HRP leads to a lower affinity for the substrate. This may be due to the reduced enzyme activity in immobilized enzyme sample. Such observations are also noted in the previous studies (Monier et al. 2010).

### Decolorization of tannery effluent using free HRP

**Figure 8:**
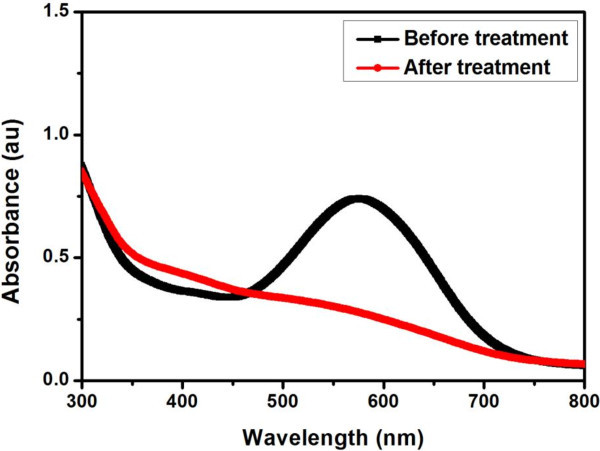
**Absorption spectra of tannery effluent before and after treatment with the free HRP.**

In order to look at the efficiency of the free HRP for degrading the dye in tannery effluent, which is a mixture of dye and chemicals such as formic acid, aromatic polymers and fat liquors generally used for making leather, we have used C.I.Acid Blue 113 in the dyeing process of goat leathers and collected the effluent. The collected effluent was suitably diluted to achieve 30 ppm concentration equivalent to the optimized dye concentration in order to treat the effluent using free HRP under optimized reaction parameters. 3 mL of the diluted tannery effluent was treated with optimized reaction parameters such as 0.08 U of free HRP, 14 μL of H_2_O_2_ for 45 min at pH 6.6 and 30°C. The UV-Visible absorption spectra recorded between 300 and 800 nm for the tannery effluent before and after free HRP treatment is shown in Figure 8. The absorbance spectrum of the tannery effluent containing the unutilized C.I.Acid Blue 113 exhibits a red-shifted absorption band with λ_max_ at 578 nm in comparison to the λ_max_ at 566 nm for pure C.I.Acid Blue 113 solution in distilled water without any chemical auxiliaries. This could be due to the fact that the tannery effluent contains not only unutilized C.I.Acid Blue 113 but also other chemicals, which can possibly interact with the dye molecule causing the red shift. The free HRP treated tannery effluent did not show any absorption band thereby demonstrating its ability to remove color from the tannery effluent. However, care should be exercised on the use of HRP in the real-time application since HRP can denature in extreme conditions such as strong acid and basic medium and high temperature (Shi et al. 2011). Although immobilized HRP can be advantageous under these extreme conditions (Alemzadeh and Nejati 2009), a semi-technical trial can be carried out before attempting industrial scale treatment.

## Conclusions

The performance of both the free and immobilized HRP depends on the reaction time, pH, temperature, HRP and H_2_O_2_ concentration. Ambient conditions such as room temperature (30°C) and near-neutral pH (6.6) were suitable for the action of HRP (0.08 U) in both the free and immobilized forms on the optimized dye concentration (30 mg/L) with the assistance of 14 μl H_2_O_2_. Free HRP has a faster reaction time (45 min) than immobilized HRP (4 h). The latter can be recycled for at least 3 times thereby demonstrating its potential for application at industrial level. The kinetic parameters for this study are also in line with the optimization experimental results. Free HRP was used for treating actual tannery effluent and the results obtained were satisfactory. This study demonstrates a feasible method for treating tannery effluent. This method could be used to achieve a sustainable and greener environment.

## Electronic supplementary material

Additional file 1: **For preliminary data and calculation for drawing kinetics of free HRP and drawing kinetics of immobilized HRP.** (DOC 104 KB)
