# Supplementary material for: Probing horseradish peroxidase catalyzed degradation of azo dye from tannery wastewater
Source: Springerplus. 2013 Jul 24;2:341. doi: 10.1186/2193-1801-2-341 (PMC3736071; doi:10.1186/2193-1801-2-341)
Supplement: Supplementary file 1 — Additional file 1: For preliminary data and calculation for drawing kinetics of free HRP and drawing kinetics of immobilized HRP. (DOC 104 KB) [file 40064_2013_422_MOESM1_ESM.doc]

**Probing horseradish peroxidase catalyzed degradation of azo dye from tannery wastewater**

### Sadhanandam Preethi, Ayyappan Anumary, Meiyazhagan Ashokkumar, Palanisamy Thanikaivelan

### Advanced Materials Laboratory, Center for Leather Apparel & Accessories Development, Central Leather Research Institute (Council of Scientific and Industrial Research), Adyar, Chennai 600 020, India

**Preliminary data and calculation for drawing Kinetics of free HRP**

ɛ=16484.265 C_32_H_21_N_5_Na_2_O_6_S_2_= 681.16144

| **Dye Substrate (mg/L)** | **S (mol/L) E-3** | **1/S E3** |
| --- | --- | --- |
| **20** | 0.029 | 34.4827 |
| **30** | 0.044 | 22.727 |
| **40** | 0.058 | 17.241 |
| **50** | 0.073 | 13.698 |

For finding V,

| **Time (mins) for 20 mg/L** | **Conc. Of substrate degraded(mol/L) E-5** |
| --- | --- |
| **15** | 1.95 |
| **30** | 2.13 |
| **45** | 2.22 |
| **60** | 2.257 |
| **75** | 2.276 |

| **Time (mins) for 30 mg/L** | **Conc. Of substrate degraded(mol/L) E-5** |
| --- | --- |
| **15** | 2.884 |
| **30** | 3.169 |
| **45** | 3.266 |
| **60** | 3.3263 |
| **75** | 3.356 |

| **Time (mins) for 40 mg/L** | **Conc. Of substrate degraded(mol/L) E-5** |
| --- | --- |
| **15** | 3.204 |
| **30** | 3.774 |
| **45** | 3.956 |
| **60** | 4.095 |
| **75** | 4.1803 |

| **Time (mins) for 50 mg/L** | **Conc. Of substrate degraded(mol/L) E-5** |
| --- | --- |
| **15** | 3.4661 |
| **30** | 3.831 |
| **45** | 4.528 |
| **60** | 4.74 |
| **75** | 4.8674 |

Equation of line for

20 mg/L Y =1.931E-5+5.26667E-8 X

30mg/L Y =2.866E-5+7.46667E-8 X

40 mg/L Y =3.155E-5+1.52667E-7 X

50mg/L Y =3.175E-5+2.47333E-7 X

Velocities 1/V

20 mg/L 1.931E-5 51786.64

30mg/L 2.866E-5 34891.84

40 mg/L 3.155E-5 31695.72

50mg/L 3.175E-5 31496.06

**Preliminary data and calculation for drawing Kinetics of immobilized HRP**

For finding V,

| **Time (mins) for 20 mg/L** | **Conc. Of substrate degraded(mol/L)** |
| --- | --- |
| **25** | 1.3E-6 |
| **85** | 9.6E-6 |
| **125** | 1.41E-5 |
| **200** | 1.9E-5 |

| **Time (mins) for 30 mg/L** | **Conc. Of substrate degraded(mol/L)** |
| --- | --- |
| **25** | 8.1E-6 |
| **85** | 1.08E-5 |
| **125** | 1.386E-5 |
| **200** | 2.05E-5 |

| **Time (mins) for 40 mg/L** | **Conc. Of substrate degraded(mol/L)** |
| --- | --- |
| **25** | 6E-6 |
| **85** | 1.75E-5 |
| **125** | 2.34E-5 |
| **200** | 3.18E-5 |

| **Time (mins) for 50 mg/L** | **Conc. Of substrate degraded(mol/L)** |
| --- | --- |
| **25** | 1.5E-5 |
| **85** | 2.22E-5 |
| **125** | 2.64E-5 |
| **200** | 3.55E-5 |

Equation of line for

20 mg/L Y =6.36258E-8+1.00564E-7 X

30mg/L Y =5.50635E-6+7.18036E-8 X

40 mg/L Y =3.77572E-6+1.462E-7 X

50mg/L Y =1.20974E-5+1.16575E-7 X

Velocities 1/V

20 mg/L 6.36258E-8 15716894.72

30mg/L 5.50635E-6 181608.51

40 mg/L 3.77572E-6 264850.15

50mg/L 1.20974E-5 82662.39
